# Photopheresis efficacy in the treatment of rheumatoid arthritis: a pre-clinical proof of concept

**DOI:** 10.1186/s12967-019-2066-1

**Published:** 2019-09-18

**Authors:** Céline Coppard, Francis Bonnefoy, Dalil Hannani, Françoise Gabert, Olivier Manches, Joel Plumas, Sylvain Perruche, Laurence Chaperot

**Affiliations:** 1 Institute for Advanced Biosciences, Université Grenoble Alpes, Inserm U 1209, CNRS, UMR 5309, 38000 Grenoble, France; 2 Etablissement Français du Sang Auvergne-Rhône-Alpes, Research and Development Lab, 29 Av Maquis du Grésivaudan, 38701 La Tronche, France; 3 Univ. Bourgogne Franche-Comté, INSERM, EFS BFC, UMR1098 RIGHT, Interactions Hôte-Greffon-Tumeur/Ingénierie Cellulaire et Génique, 25000 Besançon, France; 4 CNRS, CHU Grenoble, Grenoble INP, TIMC-IMAG, UMR 5525, Université Grenoble Alpes, 38000 Grenoble, France

**Keywords:** Collagen-induced arthritis, Extracorporeal photopheresis, Preclinical study, Autoimmunity

## Abstract

**Background:**

Despite major advances in rheumatoid arthritis outcome, not all patients achieve remission, and there is still an unmet need for new therapeutic approaches. This study aimed at evaluating in a pre-clinical murine model the efficacy of extracorporeal photopheresis (ECP) in the treatment of rheumatoid arthritis, and to provide a relevant study model for dissecting ECP mechanism of action in autoimmune diseases.

**Methods:**

DBA/1 mice were immunized by subcutaneous injection of bovine collagen type II, in order to initiate the development of collagen-induced arthritis (CIA). Arthritic mice received 3 ECP treatments every other day, with psoralen + UVA-treated (PUVA) spleen cells obtained from arthritic mice. Arthritis score was measured, and immune cell subsets were monitored.

**Results:**

ECP-treated mice recovered from arthritis as evidenced by a decreasing arthritic score over time. Significant decrease in the frequency of Th17 cells in the spleen of treated mice was observed. Interestingly, while PUVA-treated spleen cells from healthy mouse had no effect, PUVA-treated arthritic mouse derived-spleen cells were able to induce control of arthritis development.

**Conclusions:**

Our results demonstrate that ECP can control arthritis in CIA-mice, and clarifies ECP mechanisms of action, showing ECP efficacy and Th17 decrease only when arthritogenic T cells are contained within the treated sample. These data represent a pre-clinical proof of concept supporting the use of ECP in the treatment of RA in Human.

## Background

Among autoimmune diseases, rheumatoid arthritis (RA) [1] is a complex common autoimmune disease responsible for progressive disabilities due to synovial inflammation, bone and cartilage destruction associated with systemic disorders. Rheumatoid arthritis development involves environmental factors that trigger the disease in individuals with a predisposing genotype, such as some HLA DRβ polymorphisms, and it affects 0.5 to 1% of Caucasian individuals in western countries. During the course of RA, immune cells infiltrate the synovial sublining, half of them being CD4+ memory T cells, and it has been shown that autoreactive T cell frequency is correlated with disease activity [2]. Type 1 helper T (Th1) cells have long been considered as crucial in RA development, but accumulating evidence support a major role for Th17 cells in RA pathogenesis. Despite major advances in rheumatoid arthritis outcome, not all patients achieve remission, and there is still an unmet need for new therapeutic approaches [3].

Few clinical trials have assessed the therapeutic effect of extracorporeal photopheresis (ECP) in rheumatoid arthritis. In 1991, 7 patients with RA were treated by ECP, and interesting clinical improvements were reported for 4 of them [4]. clinical improvements were reported in 1993 for seven ECP-treated RA-patients [5], and in 1996, for 12 patients with psoriatic arthritis [6]. Despite these encouraging results (overall clinical improvement for approximately 50% of patients [7]), the clinical use of ECP for RA and autoimmune diseases treatment remains rare. Indeed, ECP is a cell therapy mostly applied to treat graft versus host diseases, transplant rejection and cutaneous T cell lymphomas. The therapeutic process is based on extraction of mononuclear cells by apheresis, followed by treatment of cells with 8-methoxy-psoralen (8-MOP) and exposure to ultraviolet A light. This procedure results in crosslinking of DNA pyrimidine bases in all treated cells, leading to their apoptosis. The cells are then reinfused to the patients. Depending on the disease, ECP is thought to trigger an immunomodulation either leading to immunization (in CTCL context) or immunosuppression (in GVHD or transplant rejection) [8–10].

The American council on ECP has recently published a consensus report, describing ECP as a bidirectional therapy, able to induce both immunizing and tolerizing effects [11]. ECP seems to be a safe and efficient treatment for diseases that are associated to T cell dysregulations, leading to specific long-lasting immunosuppression, making it attractive to treat autoimmune diseases [12]. The central role of T cells in RA pathogenesis, with circulating autoreactive T cells is a strong argument to propose ECP as a therapy for this disease. The lack of randomized clinical trials and the poor understanding of ECP mechanism of action clearly hinder ECP development in the context of autoimmune diseases. In order to evaluate the potential efficacy of ECP in arthritis, we used a well characterized mouse model of collagen-induced arthritis, extensively used for modeling human RA. In this paper, we show that ECP is efficient in reversing arthritis, by decreasing Th17 cells. These data represent a pre-clinical proof of concept, rationalizing the use of ECP in the treatment of RA, and provide a relevant study model for dissecting ECP mechanism of action in autoimmune diseases.

## Materials and methods

### Mice

Six- to eight-week-old male DBA/1 mice were obtained from Janvier Labs (Le Genest-Saint-Isle, France), housed at the University of Grenoble animal core facility PHTA (Plateforme de Haute Technologie Animale, agreement #C 38516 10 006 delivered by Direction Départementale de la Protection des Personnes de l’Isère), in individually ventilated cages (5 mice per cage) and fed ad libitum a standard diet and filtered water. All experiments were approved by the local ethic committee as well as French ministry of Research and Innovation, under the number: 2015061815254659. These animal experiments comply with the ARRIVE guidelines [13]. Some of the experiments have been performed at the UMR1098 animal facility (agreement #C25-056-7; Besançon, France) under #02831 project number authorization.

### Induction of collagen-induced arthritis

**Fig. 1:**
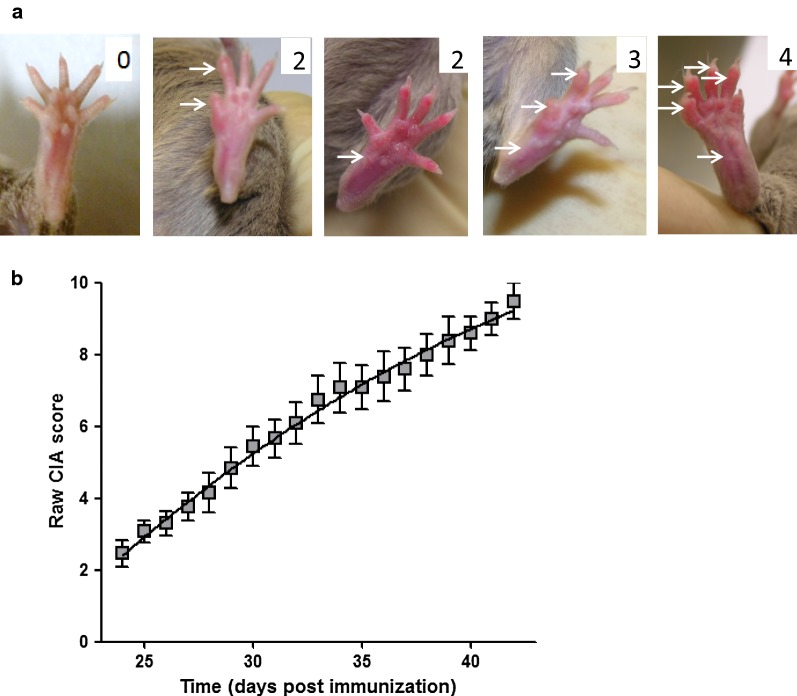
Typical clinical pattern of collagen-induced arthritis in male DBA/1 mice. Arthritis symptoms started 3 weeks after immunization. **a** The severity of arthritis was monitored by using the classical scoring system, based on paw swelling and erythema evolution. The value on each picture represents the score determined for the displayed paw. **b** Individual paw scores were summed to determine the clinical score of the mouse. Arthritis was mild at first, and became increasingly severe

DBA/1 mice were immunized by subcutaneous injection at the tail base with 100 µl of emulsion of bovine collagen type II (200 mg/ml dissolved in 0.5 M of acetic acid, MD Bioscience) and Complete Freund adjuvant (4 mg of Mycobacterium tuberculosis toxin in 1 ml of incomplete Freund adjuvant, Sigma-Aldrich), 50 µl in each side of the tail. Arthritis developed at days 21–28 after collagen immunization. The health status of the animals was carefully monitored, by daily observation of animal behavior and external physical appearance. Animals were also weighed every day. The protocol dictated that mice losing more than 20% of start weight at any time would be sacrificed, however this did not occur. No unexpected adverse event occurred. Arthritis severity was measured by visual evaluation of the paws. Each paw was scale of 0–4, where 0 = normal paw, 1 = swelling in one digit, 2 = swelling of one or more digit or mild swelling of the entire paw, 3 = moderate erythema and swelling of the entire paw, and 4 = erythema and severe swelling involving the entire paw (Fig. 1a). The clinical score for each mouse was the result of the sum of the four paws (maximum score 16). Food and water were placed directly in the cage for animals with a score higher than 8, to compensate for their mobility impairment. To take into account disease heterogeneity at the time of treatment, mice were stratified according to arthritis score and split into the different treatments groups (n = 5 mice per group). The sample size was calculated on the basis of pilot experiments, indicating that in untreated animals the mean arthritis score at day 40 post-collagen injection was 9.6 (SD = 0.88). Power calculations indicated that for a mean difference in arthritis score of 2 points to be detected with 90% power at this time point with a one-sided significance level of 5%, 5 mice in each treated group were required (calculation made with R software, epi R package). The average and standard deviation of clinical scores were similar for each group in each experiment (n = 5). To standardize the monitoring of the therapeutic effect of ECP on arthritis development, we calculated for each mouse and each day a relative CIA score, by subtracting the value of CIA score at the beginning of the treatment to the measured CIA score. Hence, on day 0, when the first injection was performed, all mice have a relative CIA score of zero. Arthritis score was measured every day, and the relative arthritis score was calculated. If arthritis progressed, the calculated CIA score was positive, and if arthritis regressed the relative CIA score was below zero.

### Induction of apoptotis by PUVA treatment

Spleens of the donor mice (arthritic or healthy mice) were harvested and mechanically dissociated. Erythrocytes were removed with a red blood cell lysis solution (eBiosciences). Spleen cells (10·10^6^ cells/ml) were treated by PUVA during 15 min at 37 °C with 8-MOP (200 ng/ml, Sigma-Aldrich) and irradiated with UV-A (365 nm, 2 J/cm^2^). Cells were washed twice with PBS. Irradiated cells (PUVA cells) were reinjected intravenously (10·10^6^ cells in 200 µl of PBS) three times every other day. Treatment was initiated when the mean arthritic score reached 7. Mice receiving arthritic mice-derived PUVA-treated-spleen cells were called «Arthritic PUVA» (A-PUVA) mice while mice receiving healthy mice-derived PUVA treated-spleen cells were called «Healthy PUVA» (H-PUVA) mice.

### Flow cytometry analysis

Apoptotic PUVA treated-T cells were stained by anti-mouse CD3-PE, Annexin V and 7AAD (AnnV-FITC/7AAD kit, Beckman Coulter) and measured 5 h after the treatment by flow cytometry. The percentage of apoptotic T cells includes early (AnnV+/7ADD−) and late (7AAD+) apoptotic T cells. For ex vivo T cell analysis, following mice harvest, spleens were collected and mechanically dissociated. Spleen cells have been homogenized and T cells were stimulated for 6 h with PMA (50 ng/ml)/Ionomycin (1 µg/ml) (Sigma) in presence of monensin (protein secretion inhibitor BD Golgistop, BD biosciences). T cells were stained extracellularly by CD3 AF488 (17A2, Biolegend), CD4-BV421 (GK1.5, Biolegend) and CD25-PECy7 (PC61.5, eBiosciences), and after fixation and permeabilization (Foxp3 Transcription Factor Staining Buffer, Invitrogen or Cytofix/cytoperm plus, BD Biosciences), intracellular cytokines and transcription factor [IL-17A-APC (TC11-18H10.1, Biolegend), IFN-γ-PE (XMG1.2, BD Biosciences) and FoxP3-PE (FJK-16s, eBiosciences)] were stained.

### Cytokine measurement

Sera of the mice were obtained after retro-orbital sampling and centrifugation (2000*g*, 10 min). Cytokines (IL-2, -4, -6, -10, -17A, IFN-γ and TNF-α) were quantified by cytometric bead array (CBA Mouse Th1/Th2/Th17 Cytokine Kit, BD Biosciences). Quantification of cytokines was performed by FACS Canto II cytometer (BD).

### Statistical analysis

Statistical analyses were performed by Prism6 software (GraphPad; La Jolla, CA, USA). Statistical significance was determined by indicated adequate tests (Mann–Whitney to compare two groups or Kruskal–Wallis test with Dunns post-test for more than two groups). To compare the different curves, a nonlinear regression was performed, and a best fit second order polynomial equation was determined for each curve (y = B0 + B1*x + B2*x^2^). These curves were then compared using two methods: the extra sum-of-squares F test, and Akaike’s information criteria. In addition, curves fitted according to the 4-parameter Baranyi model were compared using Bayesian analysis, as implemented in the function Bayescompare from the R™ software package babar (Lydia Rickett, Matthew Hartley, Richard Morris and Nick Pullen (2015). babar: Bayesian Bacterial Growth Curve Analysis in R. R package version 1.0. https://CRAN.R-project.org/package=babar). The competing hypotheses were: curves are replicates, and curves’ parameters are all different.

## Results

### ECP treatment reverses arthritis progression

**Fig. 2:**
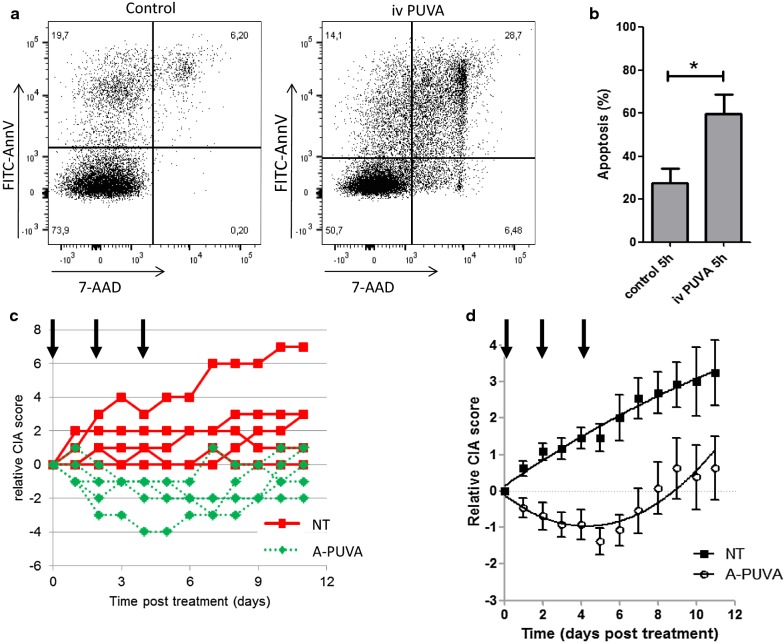
ECP treatment efficiently reverses arthritis progression. Spleen cells from arthritic mice were submitted to psoralen + UVA irradiation, resulting in their rapid apoptosis. **a** Representative flow cytometry profile of cells 5 h after ivPUVA treatment of the cells. Live cells are defined as AnnV negative 7-AAD negative cells. **b** Mean and standard deviation of 5 samples from 5 different arthritic mice spleen cells. Statistical differences between treated and untreated mice were determined by Mann and Whitney test (“*” means p < 0.05). **c** Mice with established arthritis were injected (green dotted lines) or not (red plain lines) 3 times (arrows) with ivPUVA spleen cells taken from arthritic mice. Each curve represents the relative clinical score of one mouse. **d** Mean and standard error of the mean (SEM) of relative clinical score in 13 mice per group from two different experiments. The two curves represent the calculated nonlinear regression, second order polynomial equations and are statistically different (comparison of polynomial coefficients) for untreated (black squares) and treated (white circles) mice, reflecting the different overall clinical course in treated versus untreated mice. Bayesian analysis using a Baranyi model also indicated that the two curves were different (Bayes factor > 1000)

Arthritis was induced by the injection of type II bovine collagen mixed with complete Freund adjuvant. Arthritis symptoms arose around 3 weeks after immunization. The severity of arthritis was monitored every day with the classical established scoring system, based on paw swelling and erythema evolution, as illustrated in Fig. 1a. Each paw individual score were summed to determine the clinical score of the mouse. As shown in Fig. 2b, arthritis raw score continuously grows, starting from a score of 3 at 25 days post immunization and reaching a score of 10 at 42 days post immunization.

To determine whether ECP has a beneficial effect in the treatment of arthritis, we have translated the human ECP process to adapt it to mice. In humans, ECP is an autologous procedure where blood cells are extracorporally exposed to PUVA (psoralen + UVA irradiation) before being infused back to the patient. Such procedure cannot be performed in an autologous manner in mice. Thus, we used spleen cells from an arthritic mouse (mean score = 7) as source of cells to be injected to a second arthritic mouse, following PUVA treatment.

Spleen cells from donor arthritic mice were harvested, incubated with 8-MOP and irradiated with UV-A (PUVA procedure), in the same conditions as for therapeutic ECP procedure in human. This procedure induced leucocyte apoptosis that was measured by annexin V/7-AAD labeling (Fig. 2a). Five hours after PUVA application, 60% of treated leucocytes were apoptotic (AnnV^+^7-AA^−^ cells) or underwent secondary necrosis (AnnV^+^7AAD^+^), p = 0.04(Fig. 2b). PUVA-treated spleen cells obtained from arthritic mice were immediately transferred to another group of arthritic mice. Each recipient arthritic mouse received 3 ECP treatments every other day. As shown in Fig. 2c, the relative CIA score of untreated mice increased over time, while ECP-treated mice recovered from arthritis as evidenced by a decreasing score. The analysis of 13 mice per group pooled from 2 independent experiments demonstrated the ability of ECP to reverse arthritis (Fig. 2d and Additional File 1: Fig S1a). Of note, 2 days after the third and last injection, ECP-treated mice were no longer able to control arthritis development and clinical scores began to progress again, suggesting the need to continue treating the mice to maintain therapeutic efficacy, as observed in humans. Taken together these data prove that ECP treatment can efficiently induce the regression of collagen induced arthritis in mice.

### ECP triggers Th17 decrease in vivo

**Fig. 3:**
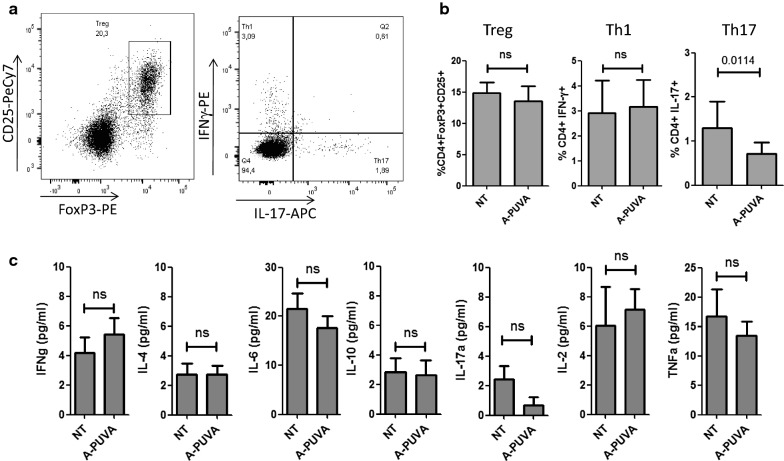
ECP treatment modifies Th17 frequency. Immune cell profile was analyzed in mice treated or not by ECP 12 days after beginning of the treatment. **a** Representative flow cytometry profile of regulatory T cells (FoxP3+/CD25+) and Th1 (IFNγ+) and Th17 (IL17+) cells labeling, on gated CD3 + CD4 + T lymphocytes. **b** Mean percentages (± standard deviation) of Treg, Th1, and Th17 in the blood of treated and non-treated mice (10 mice per groups in two independent experiments). **c** Cytokine concentrations in sera were measured by cytometric bead array. Bars represent the mean and standard deviation for 10 mice per groups in two independent experiments. Statistical differences between treated and untreated mice were determined by Mann and Whitney test

In order to gain further insights on ECP mechanism of action, we evaluated the impact of such treatment on Th17 and Th1 cells (autoimmune cells described as major players in CIA pathogenesis), as well as on Tregs (that help control disease progression), by flow cytometry analysis, as illustrated in Fig. 3a. While ECP does not affect either Tregs or Th1 cell frequencies, it significantly decreases the frequency of Th17 cells within the spleen (Fig. 3b). No differences were observed in lymph nodes (not shown).

In order to evaluate systemic inflammation, serum cytokines were also assessed. Similar levels of circulating IFNγ, IL-4, IL-6, IL-10, IL-2 and TNFα were observed between untreated and ECP-treated mice. Interestingly, serum IL-17 was also decreased under ECP treatment, although the trend did not reach statistical significance (Fig. 3c).

These data suggest that ECP treatment can modulate IL17 production by Th17 cells.

### The presence of arthritogenic T cells within the treated sample is mandatory for ECP efficacy in arthritis

In human, it is currently admitted that ECP can be efficient only in pathologies where circulating pathogenic T lymphocytes are present, thus representing a fraction of the treated cells. To test whether pathogenic T cells were also needed for ECP efficacy in CIA, we compared the therapeutic effect of PUVA-treated splenocytes originating from arthritic (A-PUVA) or healthy mice (H-PUVA). Interestingly, only PUVA-treated arthritic mouse derived-spleen cells were able to control arthritis development, while spleen cells from healthy mouse failed to do so. Statistical analyses comparing the two curves highlighted the significant difference of CIA progression in mice treated with A-PUVA spleen cells compared to mice treated with H-PUVA spleen cells or not treated, and the absence of therapeutic effect with H-PUVA spleen cells.

**Fig. 4:**
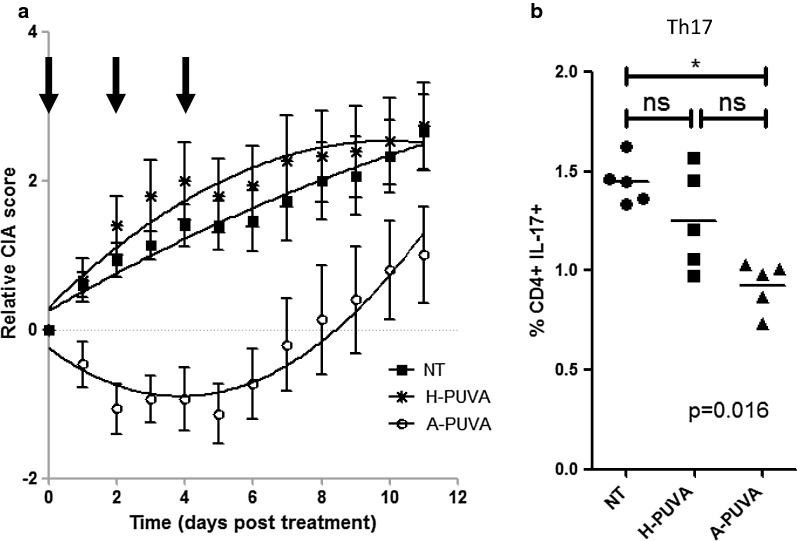
ECP efficacy relies on the presence of arthritogenic T cells in the treated sample. Mice with established arthritis were left untreated or were injected 3 times with ivPUVA spleen cells taken from arthritic or healthy mice. **a** Mean and SEM of relative clinical score in 15 mice per group from 3 independent experiments. The three curves represent the calculated nonlinear regression, second order polynomial equations and are statistically different for mice treated with ivPUVA spleen cells from arthritic mice (white circles) compared to untreated mice (black squares) or treated with ivPUVA cells from healthy mice (stars). Bayesian analysis using a Baranyi model also indicated that the A-PUVA curve was different from the two others (Bayes factor > 1000). **b** Th17 cell percentages were determined, and bars represent the mean for 5 mice per groups in one experiment. Kruskal–Wallis test showed significant differences between the 3 groups (p = 0.016) and Dunns’ post-test was used to compare the 3 groups (*p < 0.05)

In line with the clinical efficacy observed in Fig. 4a and Additional File 1: Fig S1b, only A-PUVA group displayed a decreased Th17 frequency while H-PUVA treated mice displayed a Th17 rate comparable to untreated mice (Fig. 4b). Taking together, these results demonstrate that ECP is able to trigger arthritis control and Th17 decrease only when arthritogenic T cells are contained within the treated sample.

## Discussion

The newly developed treatments based on synthetic or biologic disease-modifying anti-rheumatic drugs (DMARD) interfering with the inflammatory process [3], allow 10 to 50% of patients with RA to reach 70% improvement in the American College of Rheumatology response criteria (ACR70); their sequential use allows a good control of RA, and many patients reach remission. However, despite these recent improvements, new therapeutic strategies are needed for primary or secondary non-responder RA patients who do not reach low disease activity.

ECP can be used to treat T cell-mediated diseases, with great efficacy in various disease contexts, either leading to tolerogenic or immunogenic immune responses [11]. ECP has been recently shown to induce anticancer immunity, especially when DC differentiation is initiated during the apheresis process, while tumor cells are rendered apoptotic by the PUVA treatment [14]. On the other hand, in allogeneic T cell-mediated diseases such as GvHD, organ transplantation and T cell-mediated autoimmune diseases, ECP can have a tolerizing effect. Since RA is thought to involve Th17 autoreactive T cells, ECP could be a very promising therapeutic option for this debilitating disease. Unlike conventional immunosuppressive regimen, ECP-induced tolerance seems specific rather than systemic, and does not increase the risk of opportunistic infections or cancer development. This considerable advantage renders ECP very attractive as a therapeutic option for the treatment of autoimmune diseases, but only few clinical trials have assessed its efficacy in the treatment of autoimmune diseases. In RA, the results of published small cases series are unfortunately often controversial [9, 12], making it crucial to develop robust and easy to handle preclinical animal model to rationally evaluate ECP efficacy in this therapeutic indication. Different animal models of arthritis are currently available, each of them displaying immunologic similarities and differences with human rheumatoid arthritis [15]. We chose the model of collagen-induced arthritis in DBA1 mice to evaluate ECP efficacy and mechanism of action because this model recapitulates most clinical, histologic and immunologic features of the human disease. Indeed, in this robust model, methotrexate, anti-TNF antibodies or Abatacept (CTLA-4Ig), that are currently used to treat RA patients, have been shown to ameliorate clinical CIA scores [16–18], rendering it particularly relevant to analyze new immunomodulatory therapeutic regimen. The CIA model is probably quite stringent, since a pellet of antigen and adjuvant continuously releases the immunogenic trigger, thereby making it difficult to maintain control of the disease in the absence of treatment. In the present study, ECP could induce similar decrease in CIA scores as more established therapies in the same model [16–18], even though the procedure was initiated at a more advanced disease stage (CIA score = 7); it is unclear whether the transient symptom decrease/stabilization is due to progressing disease despite continuous immunoregulation, or if the immunological mechanisms responsible for clinical amelioration are themselves transient in nature. It is to be noted that CIA can also be initiated in non-human primates, which could prove useful to test novel therapeutic targets and for pre-clinical development. An exact replica of the autologous ECP procedure performed in humans cannot be performed in mice, but can be approximated in inbred mouse strains.

Here, our results show that ECP can induce the decrease of CIA disease scores as soon as 2 days after the first ECP-treated cell infusion, demonstrating ECP efficacy in arthritis. In humans treated with ECP in the context of GVHD, the mean time to response observed is around 1 month [19, 20], and this time is even longer in the context of cutaneous T cell lymphoma treatment [21]. In mice however, in a recent study, tumor growth was almost completely inhibited very soon after an ECP-like treatment, suggesting a rapid efficacy of ECP in rodents [14]. The differences in time to response observed in these different studies and ours may rely on differences due to the pathologies and species considered. It is noteworthy that IL-17 and Th17 play a role in early stages of CIA in mice, and that anti-IL17A antibodies can help control arthritis in CIA mice [22]. In human rheumatoid arthritis, Th17 cells frequency and interleukin-17 levels are found associated with arthritis both at the onset and the progression of the disease [23], supporting the development of IL-17 blocking agents that are currently being tested in RA [24]. In a clinical trial that used ECP to treat systemic sclerosis patients, the percentage of peripheral Th17 cells, initially high, decreased under treatment [25]. Interestingly, in our CIA model, we observed that ECP treatment triggers the decrease of IL-17 producing Th17 cells, as well as serum IL-17 cytokine, suggesting that pathogenic Th17 cells are efficiently targeted by ECP immunomodulatory action. The observed effect is significant but moderate, perhaps because this measure was performed 1 week after the last ECP treatment, at a time point where arthritis symptoms were rising again. How Th17 frequency reduction was achieved is unclear, since Th17 levels are controlled by many factors, including among many others: differentiation-inducing and expansion-inducing cytokines (e.g. IL-1b, IL-6, TGFb, IL-21, IL-23 etc.), inhibitory cytokines (e.g. IL-10, type I IFN), microbial stimulation, selective T cell death or migration. Possible but still unsubstantiated explanations may involve differential sensitivity of Th17 cells to PUVA-induced cell death, a significant reduction of their proliferation, or modulation of Th17-expanding cytokines. In a mouse model of psoriasis (K5.hTGF-b1 transgenic mice) [26], 4-week PUVA treatment suppressed the IL-23/Th17 pathway while augmenting the frequencies of Th2 and IL-10 secreting Foxp3 + Treg, in a CTLA-4 dependent manner, and the levels of many inflammatory cytokines were reduced. Whether these features also apply to the present model is not clear, as no modulation of Treg and Th1 cell populations or IL-10 was observed in the periphery in the present study, after a shorter course of treatment.

To date, ECP mechanism of action are poorly understood. In CTCL, it has been hypothesized that ECP triggers anti-tumor specific responses directed toward the tumor T cell clone [14, 27, 28], the ECP procedure being able to trigger monocyte differentiation into DC [14]. In our hands however, in a platelet-free process, ECP-treated monocytes do not to differentiate into dendritic cells, and even if their apoptosis is slower than T cell apoptosis, all monocytes end up being apoptotic after a few days [29]. In a parallel study, we hypothesized that ECP could induce immunogenic cell death [30]. Damage-Associated Molecular Patterns expression by allogeneic activated PUVA-treated T cells was evaluated, and upregulated Calreticulin expression and HMGB-1 secretion were observed [31].

On the other hand, in the context of GVHD, it has been hypothesized that ECP-treated DCs, or liver- and spleen-resident DC, massively exposed to the reinfused ECP-treated apoptotic cells, acquire tolerogenic properties and will in turn, promote immune tolerance through Treg generation, leading to disease stabilization or regression. The monitoring of regulatory T cells during ECP treatment in human brought controversial results: an increase of Treg has been observed in the context of GHVD at some time points during ECP treatment [32–35], but in CTCL [34] or lung transplant rejection [36] lower levels of circulating Tregs were observed. Usually, Treg percentages in human diseases are highly heterogeneous [37], and do not correlate with clinical responses [38], suggesting that ECP mechanism of action does not directly affect regulatory T cells frequency. In line with these observations, in our preclinical CIA model, the decrease of arthritogenic Th17 cells induced by ECP was not associated with Treg frequency modifications.

In the context of human CTCL treatment, it is currently admitted that ECP efficacy relies on the presence of circulating tumor cells. In “consensus statement update from the UK Photopheresis Society” published in 2017, ECP is recommended in CTCL patients with proven peripheral blood involvement demonstrating a peripheral blood T cell clone and/or circulating Sezary cells representing 10% to 20% of peripheral circulating lymphocytes [39]. In all other ECP indications, no evaluation of correlations between circulating pathogenic T cell presence and clinical efficacy has been performed [40], such demonstration being particularly challenging. Our data clearly demonstrate that the therapeutic effect in CIA is obtained only when ECP-treated leukocytes originate from arthritic mice, suggesting that the presence of pathogenic T cells within PUVA treated leukocyte samples is mandatory to obtain therapeutic efficacy.

Importantly, these data indicate that the treatment of a fraction of pathogenic T cells provokes a systemic control of untreated pathogenic cells, as illustrated by the decrease of Th17 cells and serum IL-17. This observation is reminiscent of T cell vaccination (TCV) experiments where autoreactive T cells were ex vivo amplified and gamma-irradiated in order to trigger their apoptosis, before being reinfused back to the patients. Such TCV strategy aims at inducing anti-clonotypic T cell responses, directed toward pathogenic T cell clones, and has shown promising clinical responses [30]. For instance, a recent Russian clinical trial evaluating the efficacy of TCV with irradiated autologous collagen reactive T-cells in RA has shown a clinical improvement in 87% of treated patients [24]. Interestingly, we have shown that activated T cells undergo faster apoptosis than resting T cells after ECP [41]. Since autoreactive T cells are in an activated state, one can speculate that they will very rapidly undergo apoptosis following ECP, emitting immunogenic-cell death-associated signals allowing dendritic cell maturation [14, 42], and will be preferentially captured to serve as a source of Ag, for priming a specific immune response by inducing efficient anti-clonotypic responses against alloreactive T cells and control systemic pathogenic T cells as hypothesized in 2015 by Hannani [30].

## Conclusion

We demonstrate here that ECP is efficient in a preclinical mouse model of RA, and should be considered as a promising therapeutic option in the course of human rheumatoid arthritis and more broadly of any T cell mediated autoimmune disease. We provide a pertinent pre-clinical in vivo model, recapitulating both human RA- and ECP-related clinical observations, paving the way for dissecting ECP mechanism of action in autoimmune disorders. We believe that the understanding of ECP mechanisms of action will help with its rational optimization and broaden its clinical applications.

## Supplementary information

**Additional file 1: Figure S1.** ECP treatment efficiently reverses arthritis progression-raw data arthritic score. **A** Mean and standard error of the mean (SEM) of raw clinical score in 13 mice per group from two different experiments corresponding to Fig. 2d. The two curves represent the calculated nonlinear regression, second order polynomial equations and are statistically different for untreated (black squares) and treated (white circles) mice, reflecting the different overall clinical course in treated versus untreated mice. **B** Mean and SEM of raw arthritic clinical score in 15 mice per group from 3 independent experiments corresponding to Fig. 4a. The three curves represent the calculated nonlinear regression, second order polynomial equations and are statistically different for mice treated with ivPUVA spleen cells from arthritic mice (white circles) compared to untreated mice (black squares) or treated with ivPUVA cells from healthy mice (stars).

## Data Availability

All data generated or analysed during this study are included in this published article

## Abbreviations

ECP: extracorporeal photopheresis
PUVA: psoralen + ultraviolet A in vitro treatment
CIA: collagen-induced arthritis
8-MOP: 8-methoxy-psoralen
CTCL: cutaneous T cell Lymphoma
GVHD: graft versus host disease
RA: rheumatoid arthritis
