# Supplementary figures and images for: Photopheresis efficacy in the treatment of rheumatoid arthritis: a pre-clinical proof of concept
Source: J Transl Med. 2019 Sep 18;17:312. doi: 10.1186/s12967-019-2066-1 (PMC6751641; doi:10.1186/s12967-019-2066-1)

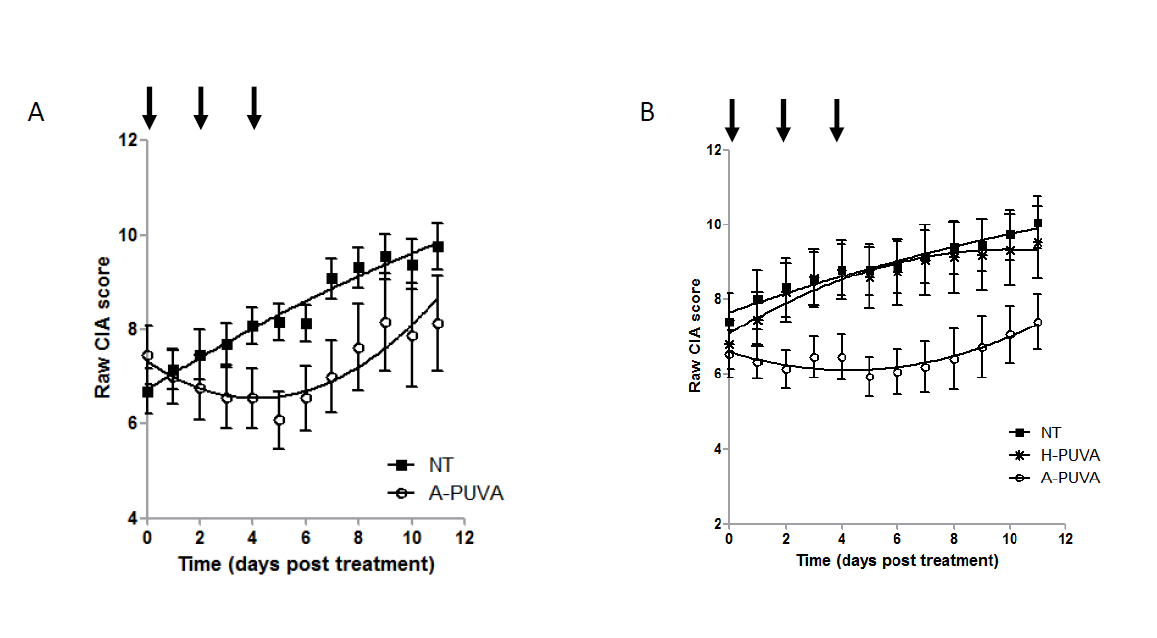

Supplement: Supplementary file 1 — Additional file 1: Figure S1. ECP treatment efficiently reverses arthritis progression-raw data arthritic score. A Mean and standard error of the mean (SEM) of raw clinical score in 13 mice per group from two different experiments corresponding to Fig. 2d. The two curves represent the calculated nonlinear regression, second order polynomial equations and are statistically different for untreated (black squares) and treated (white circles) mice, reflecting the different overall clinical course in treated versus untreated mice. B Mean and SEM of raw arthritic clinical score in 15 mice per group from 3 independent experiments corresponding to Fig. 4a. The three curves represent the calculated nonlinear regression, second order polynomial equations and are statistically different for mice treated with ivPUVA spleen cells from arthritic mice (white circles) compared to untreated mice (black squares) or treated with ivPUVA cells from healthy mice (stars). [file 12967_2019_2066_MOESM1_ESM.docx]
